# Mechanical Stretch Induces Annulus Fibrosus Cell Senescence through Activation of the RhoA/ROCK Pathway

**DOI:** 10.1155/2021/5321121

**Published:** 2021-11-19

**Authors:** Li Ning, Lei Gao, Fan Zhang, Xiaoxiao Li, Tingting Wang

**Affiliations:** ^1^Weifang People's Hospital, Weifang, Shandong, China; ^2^Department of Orthopaedics, The Third People's Hospital of Jinan, Shandong, China; ^3^Department of Orthopaedics, Eightieth Group Army Hospital of PLA Army, Weifang, Shandong, China; ^4^Shandong Medical College, Jinan, China

## Abstract

**Background:**

Intervertebral disc is responsible for absorbing and transmitting mechanical compression. Under physiological conditions, the peripheral annulus fibrosus (AF) cells are subjected to different magnitudes of transverse mechanical stretch depending on the swelling of the central nucleus pulposus tissue. However, the biological behavior of AF cells under mechanical stretch is not well studied.

**Objective:**

This study was performed to study the effects of mechanical tension on AF cell senescence and the potential signaling transduction pathway.

**Methods:**

Rat AF cells were made to experience different magnitudes of mechanical stretch (2% elongation and 20% elongation for 4 hours every day at 1 Hz) in a 10-day experiment period. The inhibitor RKI-1447 of the Rho-associated coiled-coil–containing protein kinases (ROCK) was added along with culture medium to investigate its role. Cell proliferation, cell cycle, telomerase activity, and expression of senescence markers (p16 and p53) were analyzed.

**Results:**

We found that 20% elongation significantly decreased cell proliferation, promoted G0/G1 cell cycle arrest, decreased telomerase activity, and upregulated mRNA/protein expression of p16 and p53. Moreover, the inhibitor RKI-1447 partly resisted effects of 20% elongation on these parameters of cell senescence.

**Conclusion:**

High mechanical stretch obviously induces AF cell senescence through the RhoA/ROCK pathway. This study provides us a deeper understanding on the AF cell's behavior under mechanical stretch.

## 1. Introduction

Intervertebral disc degeneration is a main cause of low back pain which often results in disability and productivity limitation around the world [1]. According to epidemiological investigation, approximately 80% adults experienced low back pain at least once throughout their lifetime [2]. Despite the heavy socioeconomic burden associated with disc degeneration, no effective treatment can restore and regenerate degenerative discs [3, 4].

The intervertebral disc suffers various kinds of mechanical load under physiological conditions [5]. The central disc nucleus pulposus (NP) tissue mainly experiences mechanical compression (dynamic or static), whereas the peripheral annulus fibrosus (AF) mainly experiences transverse mechanical stretch caused by the swelling of the central NP tissue [5]. Depending on the difference in load type, load magnitude, load duration, and cell origin, disc cells show different biological responses to mechanical stimuli [6]. According to previous studies on mechanical biology of disc cells, it is known that intracellular calcium ion and cytoskeletal remodeling are involved in the response of disc cells to mechanical stimulation, which may further regulate cell viability, gene expression, and posttranslational biosynthesis [7]. Most of these kinds of studies mainly focus on biological responses of NP cells to mechanical load, whereas mechanical biology of AF cells remains unclear.

Cell senescence is a typical cellular feature of the degenerative disc tissue, and it positively correlates with the extension of disc degeneration [8]. Senescent disc cells often have altered expression profile of catabolic cytokines and matrix degradation enzymes, which ultimately affect structural integrity [8]. Once these senescent disc cells are accumulated within the disc tissue, the cellular microenvironment is deteriorated and disc degeneration process is processed [8]. Currently, mechanical stretch is supposed to be related with AF structural deterioration during disc degeneration, such as tears and fissures [9]. In light of the positive role of mechanical overload in promoting disc NP cell senescence [10–12], it can be deduced that inhibiting AF cell senescence may be helpful to resist mechanical tension-induced AF structural destruction.

To fully understand biological effects of mechanical stretch on AF cell biology, this study investigated AF cell senescence under mechanical stretch. Cell proliferation, G0/G1 cell cycle arrest, telomerase activity, and expression of senescence-associated molecules were used to evaluate AF cell senescence. Previous studies demonstrate that the RhoA/Rho-associated coiled-coil–containing protein kinases (ROCK) signaling plays an implicit role in responding to mechanical stimuli in many cell types [13–15]. Moreover, several studies have demonstrated that the RhoA/ROCK pathway is related with intervertebral disc degeneration [16, 17]. Hence, the inhibitor RKI-1447 was used to observe the role of the RhoA/ROCK pathway in this process.

## 2. Materials and Methods

### 2.1. Rat AF Cell Isolation and Culture

The study protocol was approved by the Animal Experiment Ethical Committee of Shandong Medical Collage. In this study, thirty-five Sprague-Dawley rats (female, 7-8 weeks old) were purchased from the Animal Center of our institute. Specifically, the rats were sacrificed by air embolism via the ear vein. Then, the thoracic and lumbar discs (T11-L5) were separated under aseptic conditions. After thoroughly removing the surrounding soft tissues, the peripheral AF tissue was isolated and cut into small pieces (1 mm × 1 mm × 1 mm). And then, AF cell pellets were collected after digestion with 0.20% collagenase type I (Sigma-Aldrich, USA) at 37°C for 12-14 hours. The collected AF cells were cultured in DMEM/F12 medium supplemented with 10% fetal bovine serum (FBS, Gibco, USA) and subcultured when reaching to 80% confluence.

### 2.2. Application of Mechanical Stretch to AF Cells

The third passage AF cells were seeded on the elastic membrane that was coated with collagen I (Hangzhou Shengyou Biotechnology Co., Ltd, China), and incubated with serum-starved DMEM/F12 medium containing 1% FBS for 24 hours for synchronization. Then, the culture medium was refreshed by DMEM/F12 medium containing 10% FBS, and AF cells were stretched using a Flexercell Tension Plus System (FX-4000T, Flexcell International) at different stretch magnitudes (2% elongation and 20% elongation for 4 hours every day at 1 Hz) during a 10-day experiment period. The magnitude of 20% elongation was chosen because the mechanical stretch within this stretch does not cause alterations of AF cell phenotype [18]. The inhibitor RKI-1447 (2 *μ*M) was added along with the culture medium in the 20% elongation group to study the role of the RhoA/ROCK pathway in the effects of high magnitude of mechanical stretch.

### 2.3. Cell Proliferation Analysis

AF cell proliferation was evaluated using Cell Counting Kit-8 (CCK-8, Beyotime, China) according to the manufacturer's instructions. Briefly, after AF cells were stretched for 10 days, they were collected using 0.25% trypsin and equal AF cells (2 × 10^3^ cells per group) were seeded in a 96-well plate containing 200 *μ*L culture medium for 1, 3, and 5 days. Then, 20 *μ*L CCK-8 solution was added to the culture medium and gently mixed in each well. After incubation for 30 minutes, the absorbance value at a wavelength of 450 nm was measured using an automatic microplate reader (Thermo Fisher Scientific, USA).

### 2.4. Cell Cycle Analysis

Briefly, after mechanical stretch culture for 10 days, AF cells were collected by digestion with 0.25% trypsin without ethylene diamine tetraacetic acid (EDTA) and washed with ice-cold phosphate buffer solution (PBS). Then, they were fixed in 75% prechilled ethanol and incubated with propidium iodide (PI, 50 *μ*g/mL) solution supplemented with 100 mg/mL RNase A for 30 minutes under dark condition. Finally, they were subjected to a flow cytometry and G0/G1 cell cycle arrest was assessed using ModFit software (Verity Software House, Inc., Topsham, ME, USA).

### 2.5. Telomerase Activity Analysis

After mechanical stretch culture for 10 days, AF cells were collected and washed with PBS for two times. Then, AF cells were lysed and centrifuged to obtain the supernatant. Finally, telomerase activity (U/L) was measured according to the instructions (Mlbio, China). Briefly speaking, telomerase activity was calculated based on the standard curve and the absorbance values at 450 nm.

### 2.6. Real-Time Polymerase Chain Reaction (PCR) Analysis

Briefly, total RNA was prepared using TRIzol Reagent (Invitrogen, USA) and the first strand cDNA was synthesized using the Omniscript Reverse Transcription Kit (Qiagen, Germany). Then, real-time PCR was performed with the SYBR Green Mix (TOYOBO, Japan) on a professional machine to assess mRNA expression of p16 and p53 with 40 cycles. The primers used in the PCR were purchased from a domestic company (Sangon Biotech, Shanghai, China). The primer sequences were as follows: GAPDH: CCAGGACGCATCCACCAAGAAG (forward), GCTGCCACACGGAAGAAGACC (reverse); p16: TACCCCGATACAGGTGATGA (forward), TACCGCAAATACCGCACGA (reverse); and p53: CCTTAAGATCCGTGGGCGT (forward), GCTAGCAGTTTGGGCTTTCC (reverse). GAPDH was used as a housekeeping gene, and the relative expression of these molecules was calculated by the method of comparative 2^—△△Ct^.

### 2.7. Western Blotting Analysis

Briefly, collected AF cells were lysed on ice in RIPA buffer (Beyotime, China) for 15 minutes and then centrifuged at 15,000 × g for 15 minutes at 4°C to collect cell lysates. After measuring protein concentration using a BCA Protein Assay Kit (Beyotime, China), equal protein samples in each group were resolved in sodium dodecyl sulfate polyacrylamide gel electrophoresis (SDS-PAGE) and transferred onto the polyvinylidene difluoride (PVDF) membrane (Bio-Rad Laboratories, Hercules, CA, USA). After the PVDF membranes were incubated with primary antibodies (GAPDH: Abcam, ab8245; p16: Novus, NBP2-37740; p53: Abcam, ab26) overnight, they were incubated with secondary antibodies for 2 hours at room temperature. Finally, protein bands were visualized using the SuperSignal West Pico Trial Kit (Thermo, USA). Densitometric analysis of the protein bands was finished using the ImageJ software (National Institutes of Health, Bethesda, MD, USA).

### 2.8. Statistical Analysis

All numeric data were expressed as means ± standard deviations (SD) of three independent experiments. The one-way analysis of variance (ANOVA) was used to detect differences among groups. A *p* value < 0.05 was considered statistically significant.

## 3. Results

### 3.1. Cell Proliferation

**Figure 1:**
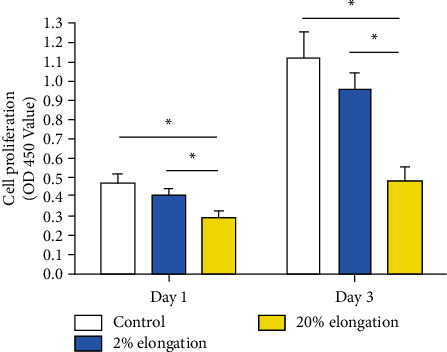
Analysis of annulus fibrosus cell proliferation under mechanical stretch. The CCK-8 assay was used to measure the OD450 value which indicates the cell proliferation potency. Data are expressed as mean ± SD (*n* = 3). ∗ represents a significant difference (*p* < 0.05) between two groups.

Senescent cells have limited ability to proliferate [19]. Results showed that the 20% elongation group has a decreased capacity to proliferate compared with the 2% elongation group and the control group on days 1 and 3. Moreover, the 2% mechanical stretch group showed a slightly decreased cell proliferation potency compared with the control group (Figure 1).

### 3.2. Cell Cycle

**Figure 2:**
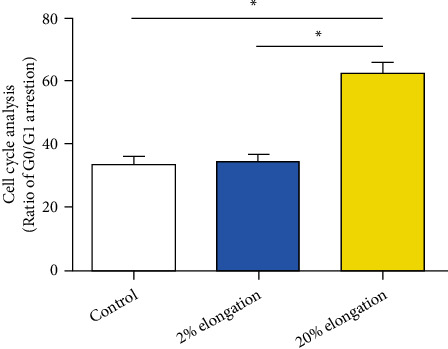
Analysis of G0/G1 cell cycle arrest under mechanical stretch. Data are expressed as mean ± SD (*n* = 3). ∗ represents a significant difference (*p* < 0.05) between two groups.

Senescent cells are often arrested in the G0/G1 cell cycle [20]. Results showed that the 20% elongation group has an increased fraction of G0/G1 cycle compared with the 2% elongation group and the control group. However, the 2% mechanical stretch group showed a similar G0/G1 cell cycle arrest compared with the control group (Figure 2).

### 3.3. Telomerase Activity

**Figure 3:**
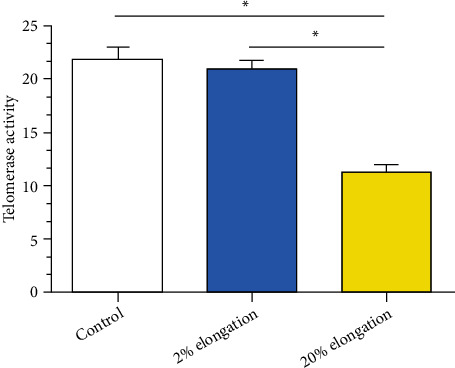
Analysis of telomerase activity under mechanical stretch. Data are expressed as mean ± SD (*n* = 3). ∗ represents a significant difference (*p* < 0.05) between two groups.

Decreasing of telomerase activity is a typical feature of senescent cells [21]. Results showed that 20% elongation group has a significantly decreased telomerase activity compared with the 2% elongation group and the control group. However, there was no statistically significant difference between the 2% elongation group and the control group (Figure 3).

### 3.4. Expression of Senescence Markers

**Figure 4:**
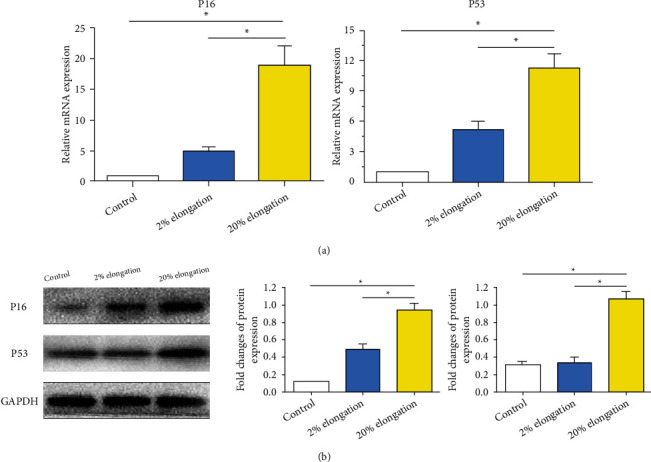
Analysis of expression of senescence-associated markers under mechanical stretch. (a) mRNA expression was evaluated by real-time PCR assay. (b) Protein expression was analyzed by western blot assay. Data are expressed as mean ± SD (*n* = 3). ∗ represents a significant difference (*p* < 0.05) between two groups.

The molecules p16 and p53 are important markers for evaluating cell senescence [21]. Results showed that both mRNA and protein expressions of them in the 20% elongation group were significantly upregulated compared with the 2% elongation group. The mRNA and protein expressions of p16 in the 2% elongation group were higher than those in the control group. For p53, an increased mRNA expression and a similar protein expression in the 2% elongation group were found compared with the control group, respectively (Figure 4).

### 3.5. RhoA/ROCK Pathway Inhibition on AF Cell Senescence under Mechanical Stretch

**Figure 5:**
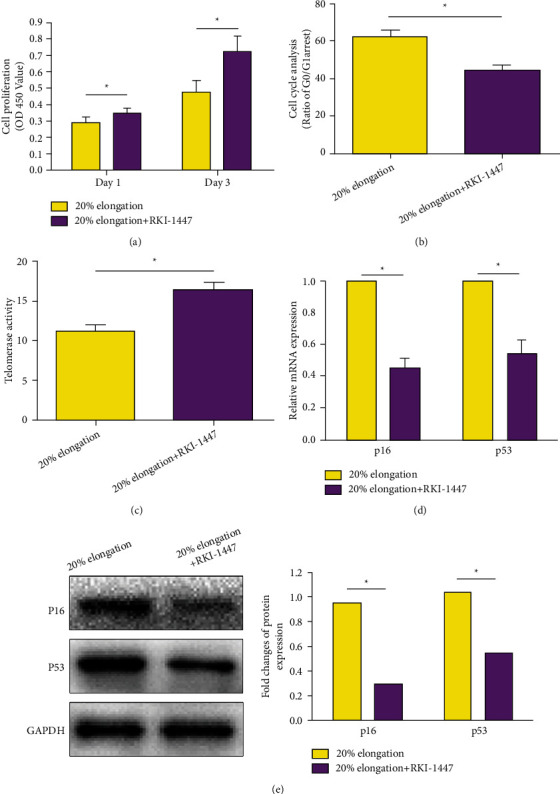
Effect of RhoA/ROCK pathway inhibition on AF cell senescence under mechanical stretch. (a–e) Results of cell proliferation, G0/G1 cell cycle arrest, telomerase activity, and mRNA and protein expression of senescence-associated molecules (p16 and p53), respectively. Data are expressed as mean ± SD (*n* = 3). ∗ represents a significant difference (*p* < 0.05) between two groups.

To evaluate the potential role of the RhoA/ROCK pathway in this process, we used the inhibitor RKI-1447 to inhibit activation of the RhoA/ROCK pathway in the 20% elongation group. Results showed that inhibition of the RhoA/ROCK pathway partly reversed cell proliferation potency, attenuated G0/G1 cell cycle arrest, increased telomerase activity, and downregulated mRNA/protein expression of senescence molecules (p16 and p53) (Figure 5).

## 4. Discussion

Disc degeneration is an important cause of low back pain. Until now, there are no effective therapies for disc degeneration because its pathogenesis remains largely unclear. Previous researches on disc degeneration have established that disc degeneration is a complex pathology affected by multiple factors [21]. Among these risk factors, mechanical load is a key factor that can initiate and accelerate disc degeneration process [22–24]. The resultant mechanical behaviors of intervertebral disc, such as alterations of disc height, fluid pressurization, tissue stiffness, and tissue flexibility, are regarded as implicit events in the initiation and progression of disc degeneration [25]. This conclusion has motivated the development of some research models to deeply investigate the disc cell's mechanobiology and the potential mechanism.

AF tissue is the peripheral region of an individual intervertebral disc which confines swelling of the central NP tissue [26]. Morphologically, AF tears and AF fissures are key features of the severely degenerative disc [9]. Increasing evidence has showed that substantial biological remodeling responsible for disc structural destruction resulted from changes in cellular bioactivity [7]. Disc cell senescence is a prominent feature within the degenerative disc tissue and found to be positively related with degree of disc degeneration [21]. Senescence-associated secretory phenotype (SASP) is a classical character of senescent cells [21], which is defined as elevation of proinflammatory cytokines and chemokines [27]. In light of the identified increase in multiple proinflammatory cytokines and matrix-degrading enzymes which can promote extracellular matrix degradation, it can be deduced that cell senescence is involved in disc degeneration process. Previously, several studies have well demonstrated that mechanical overloading facilitates NP cell senescence and induces degenerative changes similar to those in the disc degeneration process [21]. However, much less have been down to investigate the effects of mechanical load on AF cell senescence.

Senescent cells are characterized by decreased cell proliferation potency, increased G0/G1 cell cycle arrest, decreased telomerase activity, and increased expression of senescence markers (i.e., p16, p21, and p53). In this study, we used these parameters to evaluate AF cell senescence under mechanical stretch. Here, we found that the 20% elongation group significantly decreased AF cell proliferation potency, aggravated G0/G1 cell cycle arrest, decreased telomerase activity, and upregulated gene and protein expression of p16 and p53. This suggests that AF cell senescence is promoted under a high mechanical stretch. Our results are in line with those of a previous study by Zhao et al. [28].

RhoA is a member of the family of Rho GTPases, and its activity can be regulated under mechanical stimulation [21]. Rho GTPases are important for cytoskeletal dynamics and participate in cell proliferation and differentiation [21]. ROCK is a serine/threonine kinase with two isoforms (ROK*α*/ROCK-II and ROK*β*/ROCK-I) which is an important downstream effector of RhoA [21]. Activation of RhoA/ROCK signaling participates in regulating cell proliferation and proliferation [21]. When we inhibited this pathway by the specific inhibitor RKI-1447 in the 20% elongation group, we found that RKI-1447 partly reversed cell proliferation potency, attenuated G0/G1 cell cycle arrest, increased telomerase activity, and downregulated mRNA/protein expression of senescence molecules (p16 and p53). This suggests that a high mechanical stretch may accelerate AF cell senescence through activation of the RhoA/ROCK pathway.

In this study, we just investigated the role of the RhoA/ROCK pathway. However, there may be any other signaling pathways that play an implicit role in this process. More studies are needed in the future. Moreover, this study is just an in vitro study, and we did not use an in vivo animal model to further verify our conclusion, which will further enhance persuasion of the present study.

## 5. Conclusion

In conclusion, we observed the effects of mechanical stretch on AF cell senescence and investigated the role of the RhoA/ROCK pathway in this process. Our results demonstrate that a high mechanical stretch is able to accelerate AF cell senescence, and inhibition of the RhoA/ROCK pathway attenuates the effects of a high mechanical stretch on AF cell senescence. This study provides new knowledge that a high mechanical stretch promotes AF cell senescence through the RhoA/ROCK pathway.

## Data Availability

All data of this study were included in the manuscript.
